# *msaABCR* operon is involved in persister cell formation in *Staphylococcus aureus*

**DOI:** 10.1186/s12866-017-1129-9

**Published:** 2017-11-22

**Authors:** Gyan S. Sahukhal, Shanti Pandey, Mohamed O. Elasri

**Affiliations:** Department of Biological Sciences, The University of Southern Mississippi, 118 College Drive # 5018, Hattiesburg, MS 39406-0001 USA

**Keywords:** *msaABCR* operon, Persister cells, Stationary phase, Biofilm, Killing curve, Microfluidics, Gene ontology

## Abstract

**Background:**

Persister cells comprise a phenotypic variant that shows extreme antibiotic tolerance resulting in treatment failures of bacterial infections. While this phenomenon has posed a great threat in public health, mechanisms underlying their formation in *Staphylococcus aureus* remain largely unknown. Increasing evidences of the presence of persister cells in recalcitrant infections underscores the great urgency to unravel the mechanism by which these cells develop. Previously, we characterized *msaABCR* operon that plays roles in regulation of virulence, biofilm development and antibiotic resistance. We also characterized the function of MsaB protein and showed that MsaB is a putative transcription factor that binds target DNA in response to nutrients availability.

**Results:**

In this study, we compared the number of persister cell in wild type, *msaABCR* deletion mutant and the complemented strain in two backgrounds USA300 LAC and Mu50. Herein, we report that *msaABCR* deletion mutant forms significantly less number of persister cells relative to wild type after challenge with various antibiotics in planktonic and biofilm growth conditions. Complementation of the *msaABCR* operon restored wild type phenotype. Combined antibiotic therapy along with *msaABCR* deletion significantly improves the killing kinetics of stationary phase and biofilm *S. aureus* cells. Transcriptomics analysis showed that *msaABCR* regulates several metabolic genes, transcription factors, transporters and enzymes that may play role in persister cells formation, which we seek to define in the future.

**Conclusions:**

This study presented a new regulator, *msaABCR* operon, that is involved in the persister cells formation, which is a poorly understood in *S. aureus*. Indeed, we showed that *msaABCR* deletion significantly reduces the persister cells formation in all growth phases tested. Although, we have not yet defined the mechanism, we have shown that *msaABCR* regulates several metabolic, transporters, and extracellular proteases genes that have been previously linked with persister cells formation in other bacterial systems. Taken together, this study showed that inactivation of the *msaABCR* operon enhances the effectiveness of antibiotics for the treatment of *S. aureus* infections, especially in context of persister cells.

**Electronic supplementary material:**

The online version of this article (10.1186/s12866-017-1129-9) contains supplementary material, which is available to authorized users.

## Background

*Staphylococcus aureus* is an opportunistic human pathogen that colonizes more than 30% of healthy individuals worldwide [1]. This pathogen is the leading cause of nosocomial and community-associated infections, which can range from mild superficial skin infections to life-threatening infections (e.g. severe sepsis, bacteremia) and chronic biofilm-associated infections (e.g. osteomyelitis, implant-associated heart valve and native valve endocarditis) [2, 3]. Several factors contribute to the ability of *S. aureus* to be a recalcitrant pathogen. These factors include acquisition of antibiotic resistance, as well as its adaptation to diverse host environments, biofilm formation, and development of antibiotic tolerant persister cells [2, 4–9].

Treatment failures in *S. aureus* infections have been traditionally associated with antibiotic resistance; however, in the past decades, several studies have shown that drug tolerance and development of persister cells are also significant contributors [4, 8, 10–15]. Tolerance is the ability of a pathogen to survive transient exposure to lethal doses of antibiotics without a change in the minimum inhibitory concentration (MIC). Persistence is the ability of a subpopulation of the pathogen to survive lethal doses of antibiotics without any associated genetic changes [16–18]. Persister cells are dormant or phenotypic variant sub-populations that arise stochastically during the exponential growth phase, but they also arise during the stationary growth phase and within biofilms [5]. Previous study has shown that almost all stationary phase *S. aureus* cells are tolerant to conventional drugs [4], thus behaving as persister cells because of their characteristic slow and non-growing phenotype. In addition, some recent studies have shown that stationary and biofilm cells of *S. aureus* exhibit high cell density environment, deprived of nutrients, oxygen, and reduced intracellular ATP concentrations are the major determinants of persister cells [5, 19–21]. However, the mechanism for this antibiotic tolerance and persister cells formation is still not well understood, especially in *S. aureus*.

Biofilms also provide a similar conducive environment that favors the formation of persister cells by triggering pathways in response to several signals, such as dormancy within niches of biofilm, nutrient heterogeneity, quorum signaling, and oxidative stress [13, 22–30]. Studies have indicated that persister cells within biofilms are major contributors to treatment failures and recurrent infections [5, 10]. The formation of persister cells is common to most pathogens; however, the underlying mechanism remains poorly understood, especially in Gram-positive bacteria. Indeed, most studies on persister cells have been performed in *Escherichia coli*. Several pathways have been implicated in persistence, including toxin-antitoxin systems, signal transduction, SOS response and DNA repair, energy production, phosphate metabolism, membrane stress, and protein degradation [28, 30–37]. Though the persister phenotype was first described in the 1940s for *S. aureus*, very little is known about the underlying mechanism. A systematic, high-throughput screening in *S. aureus* showed that the regulation and production of metabolites are important for bacterial persistence to stresses and antibiotics [38]. Another study showed that ATP plays a crucial role in persister formation in *S. aureus* [19]. The same study found that known toxin/anti-toxin systems and stringent responses are not involved in *S. aureus* persistence [19]. Yee et al. [38] identified several genes that have been associated with formation of persister cells in *S. aureus* in the presence of rifampicin stress; however, an understanding of the detailed mechanism responsible for persister cell formation is still lacking.

The *msaABCR* operon has a critical role in biofilm development, regulation of virulence factors (proteases, pigmentation, capsule), and antibiotic resistance in *S. aureus* [39–41]. Previously, we showed that deletion of *msaABCR* operon in *S. aureus* increases protease production that leads to increased processing of the major autolysin (Atl). Uncontrolled processed major autolysin caused increased autolysis of cells and is responsible for the defective biofilm formation [41]. In another study, we showed that deletion of the *msaABCR* operon in vancomycin-intermediate *S. aureus* (VISA) strains causes significant reduction in the cell wall thickness and leads to increased susceptibility to vancomycin [42]. We have not defined the mechanism of cell wall biosynthesis regulation by the *msaABCR* operon yet; however, we have recently characterized MsaB, as a transcription activator for the capsule operon and putatively for other genes that are regulated by MsaB [39]. All our previous studies showed that *msaABCR* operon is linked with several important phenotypes like cell wall thickness, pigmentation, capsule production, and in biofilm development that are important to cope with stress environment. So, in this study, we examined the role of this operon in response to clinically relevant antibiotics stress and its role in persister cell formation. We studied persister cells formation in planktonic (exponential and stationary) and biofilm growth conditions. Since all the traditional drugs are ineffective against *S. aureus* stationary phase cells and biofilm [5, 19, 21]; we also used combination of drugs to study the persister cells under these stresses.

## Methods

### Preparation of bacterial strains and cultures

In this study, we used *S. aureus* strains USA300 LAC (Wild type, *msaABCR* deletion mutant, and complementation strains) and Mu50 (Wild type, *msaABCR* deletion mutant, and complementation strains). We used pKOR1 based allelic gene replacement method to construct a nonpolar, in-frame deletion of *msaABCR* operon from USA300 LAC strains as described previously [41]. Deletion of gene was verified by end-point and real-time quantitative PCR (qPCR), as described previously [41]. The complementation plasmid was constructed by amplifying a copy of wild type *msaABCR* operon gene with its native promoter (1788-bp fragment with complete 5′ and 3′ untranslated regions) and ligated to pCN34 (NARSA) [41]. The complementation plasmid construct was transduced into the *msaABCR* deletion mutant [41]. Overnight bacterial cultures were prepared by inoculating cells from frozen culture stocks into culture tubes containing 5 mL of freshly prepared tryptic soy broth (TSB) or Mueller-Hinton Broth (MHB, supplemented with 50 mM CaCl_2_ for daptomycin) and incubating at 37 °C with continuous shaking at 225 rpm. The overnight cultures were diluted 1:10 in fresh medium, incubated further for 2 h, and then normalized to an optical density at 600 nm (OD_600_) of 0.05 in pre-warmed 10 mL MHB in 125 conical flasks to use as the starting culture. The starting bacterial cells were grown until they reached an OD_600_ of 0.5 for exponential phase and grown for an additional 16 h for stationary cell cultures. Biofilm formation assays were performed in biofilm media (TSB supplemented with 3% sodium chloride and 0.5% glucose).

### Antibiotic susceptibility assay

All the antibiotics (daptomycin, vancomycin, linezolid, rifampicin and gentamicin) used in this study were purchased from Sigma-Aldrich, Inc. (St. Louis, MO). The minimum inhibitory concentration (MIC) for each tested antibiotic was determined by the standard broth microdilution method in MHB broth (supplemented with 50 mM CaCl_2_ for daptomycin) as previously described by the Clinical and Laboratory Standards Institute (CLSI) standards to define the susceptibility or resistance of the strains tested [43]. The MIC for each antibiotic combination was determined by the checkerboard broth microdilution method as previously described [44–46]. We used rifampicin or gentamicin as an adjuvant with daptomycin, vancomycin and linezolid and used for the combination treatment in this study. The fractional inhibitory concentration (FIC) index value, which was used to determine if the combination of antibiotics was synergistic, additive, indifferent, or antagonistic, was calculated as previously described [43, 45, 46].

### Minimum biofilm eradication concentration (MBEC) assay

The susceptibilities of biofilms (USA300 LAC, the *msaABCR* deletion mutant, and the complementation strains) to antibiotics were tested using a static MBEC 96 biofilm assay as described in the manufacturer’s manual (MBEC™-HTP assay, Innovotech Inc.) [47]. Briefly, an overnight culture of each bacterial strain was diluted to an absorbance value of 0.05 at OD_600_ in fresh biofilm media and inoculated (200 μl) onto the MBEC plate. The biofilm was allowed to grow at 37 °C on the pegs under shaking conditions (150 rpm) for 24 h. After 24 h incubation, three pegs for each strain tested were harvested to assess the biofilm. The remaining pegs were then transferred to a plate that contained 10×, 20×, 40×, or 80× MIC of tested antibiotics concentration. The plate also contained a set of control experiment where the biofilm is allowed to grow in media without antibiotics. The biofilms were further incubated at 37 °C under shaking conditions (150 rpm) for 24 h. After incubation, the pegs were rinsed in phosphate-buffered saline (PBS) to remove non-adherent bacteria, transferred to a new plate containing sterile PBS, and sonicated to remove adherent bacteria. Aliquots (100 μl) of appropriately diluted samples then were plated on tryptic soy agar (TSA) and incubated overnight at 37 °C prior to enumeration. All experiments were performed in triplicate and repeated three times.

### Persister assays

Exponential phase cells were prepared as described above. When an OD_600_ reached 0.5, 100 μL of each culture was removed, serially diluted in 900 μL of 1× phosphate buffer saline (PBS) and plated for colony forming units (CFUs) for the initial count. The remaining cultures were individually challenged with daptomycin (10 μg/ml; 10× MIC), vancomycin (25 μg/ml; 40× MIC), linezolid (50 μg/ml; 10× MIC), gentamicin (20 μg/ml; 4× MIC) and rifampicin (2.4 μg/ml; 40× MIC) and further incubated at 37 °C with continuous shaking (225 rpm). We used the minimum concentration of each antibiotic that showed similar killing kinetics to that of higher concentrations of the respective antibiotic. At designated time points of antibiotic exposure, 100 μL of cells was removed, washed with 900 μL of 1X PBS and plated for CFUs counts after 24 h of incubation. Stationary phase cells were prepared as described above. Similar to the exponential phase, 100 μL of the culture was used for the initial CFUs count while remaining was challenged with antibiotics daptomycin (80 μg/ml, 80× MIC), vancomycin (125 μg/ml, 200× MIC), linezolid (100 μg/ml, 20× MIC), rifampicin (4.8 μg/ml, 80× MIC) and gentamicin (25 μg/ml, 5× MIC). Accordingly, persister assay was performed in exponential and stationary phase culture with combination of the antibiotics. The concentrations of antibiotics used in single and combination are listed in Additional file 1: Table S1 and Additional file 2: Table S2. All the experiments were performed at least in three biological replicates.

To confirm whether the isolated persister cells show similar behavior during the antibiotic treatment, the persister cells were harvested and regrown in fresh media and again treated with antibiotics. We chose vancomycin and vancomycin/rifampicin to represent the single and combination antibiotic experiments respectively. We harvested the persister cells fractions after 48 h post-treatment with vancomycin and 72 h post-treatment with vancomycin/rifampicin. The cells were washed in 1X PBS, inoculated in MHB, regrown and exposed subsequently to the respective antibiotics. CFUs were counted and line graph was plotted to observe whether the re-treatment assays show the identical biphasic killing as the initial experiments. This re-treatment experiment was performed consecutively for 4 times.

### Catheter based-biofilm Persister assays

An in vitro catheter-associated model of biofilm formation was used to perform persister assay under biofilm conditions as previously described [48]. The concentrations of antibiotics (individual and combined) tested for this assay are listed in Additional file 3: Table S3. In brief, 1-cm segments of fluorinated ethylene propylene (FEP) catheters (14 gauge; Introcan Safety, B. Braun, Bethlehem, PA, USA) were cut in half with sterile scissors and then placed in the 24-well microtiter plate and coated overnight with 20% human plasma (Sigma-Aldrich, Inc., St. Louis, MO). The catheters (3 per well) were transferred to wells containing 2 mL of biofilm medium with respective test strains (USA300 LAC, *msaABCR* deletion mutant, and the complementation strain) normalized to an OD_600_ of 0.05. The catheters were further incubated at 37 °C with shaking for 24 h, the catheters were removed, rinsed in PBS to remove non-adherent bacteria, and placed into fresh biofilm medium supplemented with 2.5 mM CaCl_2_ with or without the indicated amounts of antibiotics, and incubated for up to four days. Each day, catheters (*n* = 3) were recovered, and the spent medium was replaced with fresh medium (containing 2.5 mM CaCl_2_ with or without the indicated amounts of antibiotics). The procedure continued till day 4. To enumerate the surviving cells after antibiotic treatment, each day, the catheters (3 per day) were harvested to enumerate the number of CFUs per catheter. The catheters were rinsed with 1× PBS to remove non-adherent bacteria and placed into a test tube containing 2 mL of sterile 1× PBS, after which they were sonicated to remove adherent bacteria. Aliquots (100 μl) of appropriately diluted samples were then plated on TSA and incubated overnight at 37 °C prior to enumeration. All experiments were performed in triplicate and repeated three times.

### Bioflux based – Biofilm persister assay

*S. aureus* biofilm development was assessed using a BioFlux200 microfluidic system (Fluxion Biosciences, Inc., San Francisco, CA, USA) as described previously [49]. Using BioFlux200 24-well plates, the biofilm growth channels were first coated overnight at 4 °C with human plasma. The channels were then primed with pre-warmed (37 °C) biofilm media; excess TSB was aspirated carefully from the output and input wells. To seed the channels with bacteria, the output wells were replaced with 200 μl of fresh overnight-cultured *S. aureus* cells diluted to an OD_600_ of 0.1 and pumped into the channels at 2.0 dynes/cm^2^ for 7 s. The cells were then allowed to attach to the channel surface of the plate for 1 h at 37 °C. The excess inoculum was aspirated from the output well, and 1.5 mL of biofilm media was added to input well A. Biofilm media supplemented with 50 mM CaCl_2_ and appropriate antibiotics was added to input well B. Input well A was pumped for 12 h at the rate of 0.6 dyne/cm^2^, and input well B was maintained to a pressure of 0.0125 dyne/cm^2^ to withstand the backflow of media. The pump pressure was reversed after 12 h to assess the effect of the antibiotics on the preformed biofilm under continuous flow. Bright-field images were acquired in 10- or 20-min intervals for 2 days using an inverted microscope (Leica DM IL LED Fluo Inverted Microscope; Leica Microsystems) under 5X magnification. For this assay, we tested the same concentrations of the antibiotics as were described above for the in vitro catheter-based biofilm assay (Additional file 3: Table S3). All the experiments were repeated three times.

### Quantification of acquired BioFlux biofilm images

Using BioFlux Montage software (Fluxion Biosciences, Inc.), the bright-field images were analyzed as previously described by Moormeier et al. [49]. A threshold was set to include all light images (biofilm cells) using the Threshold tool and Slider tool. The percentage of biofilm coverage was calculated as the total percentage of area coverage by the biofilm and was plotted against the time frame.

### RNA isolation and RNA sequencing

Total RNA for RNA sequencing (RNA-seq, HiSeq 2000) was isolated from cells using a Qiagen RNeasy Maxi column (Qiagen, Valencia CA), as previously described in Sahukhal & Elasri [41]. Briefly, overnight cultures of *S. aureus* were diluted to an OD_600_ of 0.05 in TSB and incubated at 37 °C with shaking (200 rpm) until they reached an OD_600_ of 1.5. The cells were first treated with RNA protect (Qiagen, Valencia CA) and were further proceeded for the RNA extraction. To isolate total RNA under biofilm conditions, the USA300 LAC and *msaABCR* deletion mutant’s biofilm were first grown in flow cells (Stovall Life Science, Greensboro, NC), as previously described in Sahukhal et al. [40]. The biofilm cells were harvested, treated with RNA protect (Qiagen) and were further processed for the RNA extraction. The concentration and the quality of total RNA was determined by Nanodrop spectrometer readings, as well as using a Bioanalyzer (Agilent). Only those RNA samples that have an A_260_/A_280_ ratio of ~2.0 and an RNA Integrity number (RIN) value of ≥9.7 were used for further processing. The total RNA was processed using MICROBExpress™ Kit (Ambion, AM1905) to enrich bacterial mRNA by removing the 16S and 23S ribosomal RNA from the total RNA as instructed in the MICROBExpress™ kit life technologies protocol. The concentration and the quality of enriched RNA was determined by using a Bioanalyzer (Agilent). The RNA was stored at −80 °C and shipped to Beckman coulter genomics center in dry-ice for RNA-sequencing using Illumina- HiSeq 2000. The data generated form the HiSeq200 was aligned to NC_007793.fa (*Staphylococcus aureus subsp. aureus* USA300_FPR3757, complete genome) by using Tophat-2.0.8b [50, 51]. The transcript level counts were calculated and FPKM normalized using Cufflinks-2.1.1 [52, 53]. An FPKM filtering cutoff of 1.0 was used to determine expressed transcripts and differential expression of genes in the *msaABCR* deletion mutant compared to wild type USA300 LAC was computed using Cuffdiff. All the genes that are differentially expressed greater than 3-fold were considered significant and were used for further GO enrichment analysis.

### Comparative gene ontology analysis

As a part of functional genomic analysis of our RNA-seq transcriptomics data, we performed gene ontology (GO) classification of differentially expressed genes (≥ 3-fold). We used freely available web-based GO analysis tool (Comparative GO, [54]) to better understand the differentially expressed genes in terms of biological pathways involved so that we can select some important genes for further analysis.

### Statistical analysis

All statistical analyses to test significance in this study were done by using OriginPro software (Originlab, Northampton, MA). The statistical significance level 0.05 was set as cutoff values while performing the statistical analyses. The data obtained from CFU experiments under planktonic growth phase (exponential- and stationary-) were analyzed using Student’s t-test. All the results from the biofilm experiments were analyzed using Student’s t-test and one-way ANOVA followed by posthoc Tukey’s test.

## Results

### The *msaABCR* deletion mutant show no defect in growth and antibiotic susceptibility

To study the role of the *msaABCR* operon in the *S. aureus* persister cells formation, we first examined if the deletion of *msaABCR* operon has any growth defect under planktonic conditions. Our results showed that the *msaABCR* deletion mutant has no growth defect in both exponential and stationary growth phases in nutrient rich media like MHB (Additional file 4: Figure S1). Therefore, observed difference in CFUs between the wild type USA300 LAC and the mutant strains in presence of antibiotics are due to killing and not growth defects in the mutant. Prior to conducting persister assays, we first determined the susceptibility of the *msaABCR* deletion mutant to the antibiotics under both planktonic and biofilm growth conditions. In this study, we focused on clinically relevant antibiotics (daptomycin, vancomycin, linezolid, rifampicin, and gentamicin) that are used for the treatment of serious MRSA infections as discussed in the Clinical Practice Guidelines by the Infectious Diseases Society of America [55]. We used standard microbroth dilution method and the CLSI standards to define the antibiotic susceptibility or resistance of the strains tested [43]. We found that deletion of the *msaABCR* operon did not influence the susceptibility of *S. aureus* to daptomycin, linezolid, or gentamicin. However, the deletion of the *msaABCR* operon resulted in a two-fold decrease in MIC to rifampicin and vancomycin (Table 1).

**Table 1:** Determination of the Minimum Inhibitory Concentration (MIC) of antibiotics

Antibiotics tested	Minimum inhibitory concentration (μg/mL)		
	USA300 LAC	*msaABCR*	Complementation
Daptomycin	1	1	1
Rifampicin	0.06	0.0312	0.06
Vancomycin	0.625	0.312	0.625
Linezolid	5	5	5
Gentamicin	5	5	5

**Fig. 1:**
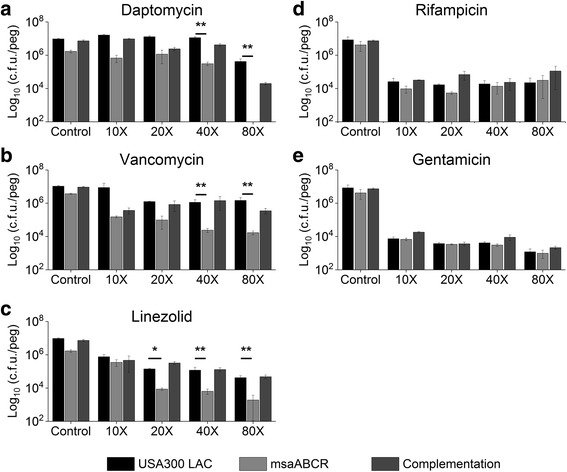
Antibiotic susceptibility in biofilm. USA300 LAC, *msaABCR* deletion mutant, and the complementation strains biofilm were grown on MBEC™-HTP plates and treated overnight with 10X to 80X MIC of **a.** daptomycin, **b.** vancomycin or **c.** linezolid. These results represent the means of three independent experiments performed in triplicate. Control in each experiment represents the biofilm grown in regular biofilm media without antibiotics. Error bars represent SE. Student’s t-test and one-way ANOVA followed by Tukey’s test (OriginPro) was used to compare the results of the wild-type to mutants (* *p*-value <0.05, ** *p*-value <0.005)

**Fig. 2:**
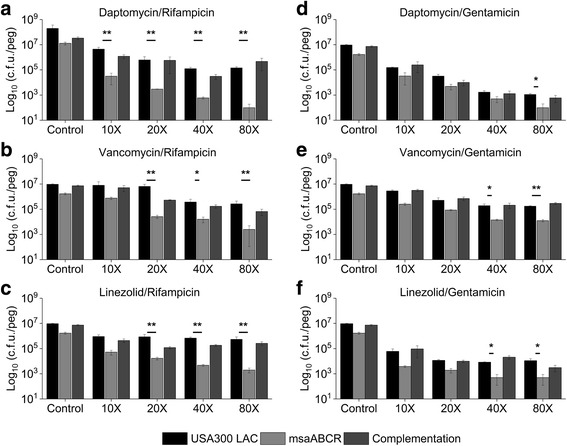
Susceptibility of *S. aureus* biofilm to combinations of antibiotics. Strains USA300 LAC, *msaABCR* deletion mutant, and the complementation biofilm were grown on MBEC™-HTP plates and treated overnight with antibiotic combinations: **a.** daptomycin/rifampicin, **b.** vancomycin/rifampicin, **c.** linezolid/rifampicin, **d.** daptomycin/gentamicin, **e.** vancomycin/gentamicin, or **f.** linezolid/gentamicin. Antibiotics amount used are multiples of the combined MIC of the combination. These results represent the means of three independent experiments performed in triplicate. Control represents biofilm grown without antibiotics. Error bars represent SE. Student’s t-test and one-way ANOVA (OriginPro) followed by Tukey’s test was used to compare the results of the wild-type to mutants (* *p*-value <0.05, ** *p*-value <0.005)

We also tested the impact of antibiotic treatment within biofilm on USA300 LAC, *msaABCR* deletion mutant, and the complementation strain by using standard 96-well plate MBEC™-HTP assay. The wildtype USA300 LAC, *msaABCR* deletion mutant, and complementation strains colonized the MBEC pegs at a rate of 1.3 × 10^7.^, 5.83 × 10^6^, and 1.67 × 10^7.^ CFUs per peg, respectively after 24 h (Additional file 5: Fig. S2). These 24 h grown biofilms were then treated with daptomycin, rifampicin, vancomycin, linezolid, or gentamicin at a range of 10× to 80× MIC in 96-well plates. Antibiotic concentrations greater than 80× MIC were not used as these are beyond the concentrations used in clinical settings. Several antibiotics significantly affected the biofilm grown on the MBEC pegs after 24 h treatment in a dose dependent manner (Fig. 1 and Fig. 2). Specifically, the biofilm of the *msaABCR* deletion mutant was significantly affected when treated with daptomycin, vancomycin, or linezolid relative to that of the wildtype USA300 LAC and the complementation strains (Fig. 1). The *msaABCR* deletion mutants’ biofilm was undetectable when treated with 80 μg/mL (80× MIC) of daptomycin (Fig. 1a). Likewise, the mutant’s biofilm was reduced by 58- to 87-fold and from 2- to 22-fold when it was treated with vancomycin and linezolid, respectively (Fig. 1b and c). The difference in CFUs observed in the mutants’ biofilm is statistically significant when analyzed using Student’s T-test. The statistics were further confirmed by one-way ANOVA followed by post-hoc Tukey’s HSD test. However, rifampicin and gentamicin treatment did not have any significant effect on the *msaABCR* deletion mutant relative to wildtype. For control, we added biofilm media without antibiotics to continue the biofilm growth. Under control condition, the *msaABCR* deletion mutant showed a 5.64-fold reduction in its ability to form biofilm relative to wildtype (Fig. 1). These results showed that although there is no clear-cutoff MBEC values for all the strains tested, the *msaABCR* deletion mutants’ biofilm was significantly affected by antibiotic treatment.

### The *msaABCR* deletion mutant showed increased susceptibility towards antibiotics with rifampicin as an adjuvant

Combination of antibiotics therapy not only enhances the efficacy of treatment [56–59] but also reduces the incidence of resistance against monotherapy [60]. In clinical practice, rifampicin or gentamicin were used as an adjuvant therapy to daptomycin, vancomycin, or linezolid [55, 59, 61]. Therefore, we assessed the impact of antibiotic combinations under both planktonic and biofilm conditions by using in-vitro checkerboard microbroth dilution method. We calculated the FIC index for each combination as defined by the *Antimicrobial Agents and Chemotherapy* journal definitions of synergy (FIC index of ≤0.5) and antagonism (FIC index of >4.0) [62]. Our result showed that in all three combinations of antibiotics that included rifampicin showed synergistic activity with FIC index values of less than 0.05 on planktonic cells (Tables 2 and 3). The combination of rifampicin and daptomycin had the greatest synergistic activity with an FIC index of 0.176 against the wildtype strain and an FIC index of 0.079 against the *msaABCR* deletion mutant (Tables 2 and 3). When gentamicin was used as an adjuvant, we did not observe any synergistic activity, except in the case of the daptomycin/gentamicin combination which showed synergistic activity against wildtype (Tables 2 and 3).

**Table 2:** MIC of in vitro combinations of antibiotics by the checkerboard broth microdilution method

	Minimum inhibitory concentration (μg/mL)		
	USA300 LAC	*msaABCR*	Complementation
DAP / RIF	0.0312 / 0.0078	0.0156 / 0.0039	0.0312 / 0.0078
VAN / RIF	0.156 / 0.0075	0.039 / 0.0075	0.156 / 0.0075
LIN / RIF	0.156 / 0.0037	0.156 / 0.00094	0.156 / 0.0037
DAP / GEN	0.0195 / 1.25	0.312 / 1.25	0.0195 / 1.25
VAN / GEN	0.312 / 1.25	0.312 / 1.25	0.312 / 1.25
LIN / GEN	0.3125 / 2.5	0.039 / 2.5	0.3125 / 2.5

DAP: daptomycin, VAN: vancomycin, RIF: rifampicin, LIN: linezolid, GEN: gentamicin

**Table 3:** Fractional Inhibitory Concentration (FIC) values for combined antibiotics as defined by CLSI standards

FIC Index	USA300 LAC			*msaABCR*			Complementation		
	FIC	FIC range	INT	FIC	FIC range	INT	FIC	FIC range	INT
DAP / RIF	0.176	0.162–0.193	SN	0.079	0.07–0.08	SN	0.176	0.162–0.193	SN
VAN / RIF	0.354	0.31–0.375	SN	0.27	0.187–0.375	SN	0.35	0.31–0.37	SN
LIN / RIF	0.246	0.18–0.315	SN	0.13	0.002–0.3	SN	0.25	0.18–0.32	SN
DAP / GEN	0.346	0.269–0.437	SN	0.54	0.52–0.56	AD	0.346	0.269–0.437	SN
VAN / GEN	0.87	0.75–1.0	AD	0.87	0.75–1.0	AD	0.87	0.75–1.0	AD
LIN / GEN	0.54	0.51–0.56	AD	0.511	0.507–0.515	AD	0.54	0.51–0.56	AD

DAP: daptomycin, VAN: vancomycin, RIF: rifampicin, LIN: linezolid, GEN: gentamicin, INT: interpretation; SN: synergy (FIC ≤ 0.5); AD: additive (> 0.5 FIC ≤ 1.0); IN: indifferent (1.0 > FIC ≤ 4.0); AN: antagonistic (FIC > 4.0)

We also observed that the antibiotic combinations were effective against biofilms in a dose-dependent manner (Fig. 2). We did not observe any clear cutoff MBEC value for the combined antibiotic treatment; however, the *msaABCR* deletion mutant biofilm was significantly reduced relative to the wildtype biofilm, and this reduction was far more significant than the corresponding comparison in the absence of antibiotics (5.64-fold). When rifampicin was combined with daptomycin, vancomycin, or linezolid, the *msaABCR* deletion mutant biofilm was reduced by 142- to 619-fold, 10- to 246-fold, or 17 to 270-fold, respectively, relative to wildtype biofilm (Fig. 2a, b, and c). In contrast, the *msaABCR* deletion mutant biofilm showed a reduction from 3.4- to 11-fold, 5.9- to 14-fold, and 6- to 22-fold in biofilm formation when gentamicin was combined with daptomycin, vancomycin, or linezolid, respectively (Fig. 2d, e, and f). This is particularly interesting since combinations that included gentamicin did not have any synergistic effects on the *msaABCR* deletion mutant under planktonic conditions (Table 3). These results indicate that antibiotic combinations that include rifampicin as an adjuvant are significantly more effective against the *msaABCR* deletion mutant in both biofilm and planktonic growth conditions relative to wildtype and the complementation strains. The difference in CFUs observed in the mutants’ biofilm is statistically significant when analyzed Students T-test. The statistics were further confirmed by one-way ANOVA followed by post-hoc Tukey’s HSD test.

### The *msaABCR* operon deletion mutant is defective in persister cells in planktonic growth

**Fig. 3:**
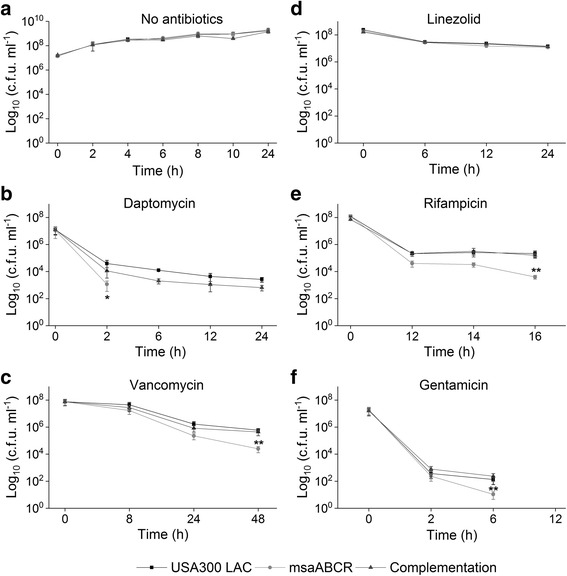
Persister cells in exponential growth phase. Strains USA300 LAC, *msaABCR* mutant and the complementation were grown to exponential growth phase. Strains were then exposed to high concentration of antibiotics: **a.** no antibiotic control, **b.** daptomycin (10 μg/ml, 10× MIC), **c.** vancomycin (25 μg/ml, 40× MIC), **d.** linezolid (50 μg/ml, 10× MIC), **e.** gentamicin (20 μg/ml, 4× MIC), and **f.** rifampicin (2.4 μg/ml, 40× MIC). Time 0 h represents antibiotic exposure point. These results represent the means of three independent experiments. Error bars represent SE. Student’s t-test (OriginPro) was used to compare the results of the wild-type to mutants at respective designated time points (* *p*-value <0.05, ** *p*-value <0.005)

In *S. aureus*, the rate of persister cell formation is dependent on growth phases [13, 19, 21]. Studies have shown that the majority of cells at stationary growth phase are slow- or non-growing and show characteristics that are similar to those of persister cells [19, 21]. Therefore, we sought to examine the impact of *msaABCR* operon-deletion in persister cell formation in both exponential and stationary growth phases by subjecting the cells to different antibiotic stress (Additional file 1: Table S1, Additional file 2: Table S2, and Additional file 3: Table S3). Under conditions without antibiotics, the *msaABCR* deletion mutant has no observable growth defect (exponential or stationary) relative to wild type USA300 LAC (Additional file 4: Figure S1a and S1b). However, the *msaABCR* deletion mutant was defective in persister cells in most of the antibiotics tested during exponential growth phase. Daptomycin (10×) rapidly killed majority of all the cells tested within 2 h of treatment. The persister plateau was observed in USA300 LAC and the complementation strains after 6 h post treatment till 24 h, however no visible CFU were detected in the *msaABCR* deletion mutant post 2 h treatment till 24 h, suggesting a defect in the formation of persister cells by the mutant in presence of daptomycin (Fig. 3b; Table 4). Likewise, the numbers of persister cells was decreased by 55-fold relative to the wildtype within 16 h of rifampicin treatment while 23-fold reduced persister cells were observed with vancomycin treatment for 48 h (Fig. 3e and c; Table 4). But, the *msaABCR* mutant formed similar proportion of persister cells as that of wild type strains with linezolid treatment for upto 72 h (Fig. 3d; Table 4). Gentamicin showed the highest killing rate forming persister plateau from 2 to 6 h, where the *msaABCR* deletion mutant formed 12.5-fold reduced persister cells relative to wild type, however, no visible CFU were observed post 6 h treatment in all the strains tested (Fig. 3f; Table 4).

**Table 4:** Comparison of the rate of persister cell formation between the *msaABCR* deletion mutant and wildtype USA300 LAC

Fold change in persister cells formation (mutant vs. wildtype)			
Antibiotics	Exponential Growth phase	Stationary Growth phase	Biofilm
DAP	UD**	- 2.15	- 747.11**
VAN	- 23.82**	- 1.40	- 2.8X10^4^**
RIF	- 54.16**	- 1.66	- 19.56
LIN	- 1.18	- 44.67**	- 14.24**
GEN	- 12.50**	UD**	- 2.33
DAP + RIF	- 25.03**	- 23.02**	UD**
VAN + RIF	- 96.12**	- 4.04	- 32.35**
LIN + RIF	1.47	- 3.35	- 4.32
DAP + GEN	- 55.30*	- 53.88**	- 0.73
VAN + GEN	- 13.23**	- 0.88	- 406.66**
LIN + GEN	+ 14.43**	- 9.43**	- 37.50**

DAP: daptomycin, VAN: vancomycin, RIF-rifampicin, LIN: linezolid, GEN: gentamicin, UD: undetectable persister cells in the *msaABCR* deletion mutant

Statistically significant (* *p*-value <0.05, ** *p*-value <0.005): The difference in CFUs observed in the mutant cells Vs wild type cells was analyzed by students T-test to measure the statistical significance. All the CFUs experiments that involved the biofilm cells were analyzed by one-way ANOVA followed by post-hoc Tukey’s HSD test to measure the statistical significance

**Fig. 4:**
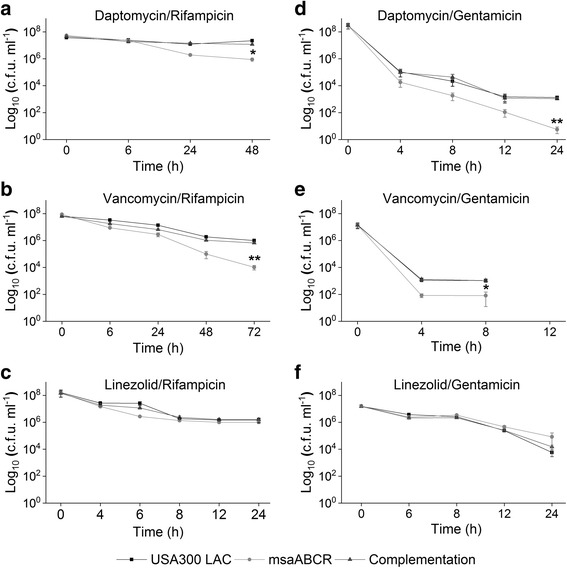
Persister cells in exponential growth phase in presence of combined antibiotic treatment. The exponentially growing cells (USA300 LAC, *msaABCR* mutant and the complementation) were treated with combined antibiotics. **a.** daptomycin/rifampicin (40× MIC), **b.** vancomycin/rifampicin (20× MIC), **c.** linezolid/rifampicin (20× MIC), **d.** daptomycin/gentamicin (20× MIC), **e.** vancomycin/gentamicin (20× MIC), and **f.** linezolid/gentamicin (20× MIC). Student’s t-test (OriginPro) was used to compare the results of the wild-type to mutants at respective designated time points (* *p*-value <0.05, ** *p*-value <0.005). These results represent the means of three independent experiments. Error bars represent SE

We also tested the effect of antibiotic combinations on the persister cell formation during the exponential growth phase. Our results show that antibiotic combinations: daptomycin/rifampicin, vancomycin/rifampicin, and daptomycin/gentamicin significantly reduced the persister cells (>10 fold) in the *msaABCR* deletion mutant relative to the wildtype (Fig. 4a, b, and d; Table 4). Vancomycin/gentamicin combination also significantly reduced the persister cells in the mutant relative to its wild type and complementation strains, however, the persister plateau was observed from 4 to 8 h treatment, and no detectable CFU were observed post 8 h treatment (Fig. 4e). Interestingly, the *msaABCR* deletion mutant showed 14-fold increased CFUs relative to wild type in response to linezolid/gentamicin combination, but we did not observe peculiar persister plateau fraction until 24 h of treatment (Fig. 4f; Table 4).

Our persister cells assay, during the exponential growth phase, in presence of individual as well as combination of antibiotics stress showed different killing kinetics and formed their characteristic persister plateau. Previous studies have shown that regular as well as persister cells of *S. aureus* are comprised of physiologically heterogeneous groups [63, 64]. This heterogeneity of fitness resulted in variety of killing curves, such that in our study, we observed biphasic killing trajectories in some of the drugs tested while the others were not as pronounced as the former. For instance, daptomycin in exponential phase, rapidly killed wild type, *msaABCR* deletion mutant and complementation strains cells within 2 h, thus sorting for highly robust persister cells. We did not observe detectable CFUs in the *msaABCR* mutant until 24 h of exposure, while 3 log of wild type and complementation cells remained viable forming a persister plateau. Similarly, pronounced biphasic killing was observed in gentamicin treatment, which killed 5 log of cells of all the strains within two hours. Steady number of CFUs were observed for wild type and complementation strains for 6 h of gentamicin treatment, however, decreasing number of CFUs count in the *msaABCR* mutant demonstrate the presence of less tolerant persister cells in the mutant. The killing curve in vancomycin treatment was not as pronounced as the formers. Moreover, the steady decreasing number of CFUs count in the *msaABCR* mutant after 24–48 h of treatment shows the presence of less tolerant persister cells in the mutant relative to wild type and the complementation strains that showed the presence of robust persister cells. Similarly, robust persister cells were isolated in wild type and complementation strains within 12–16 h of rifampicin treatment, while mutant cells only formed transient persister plateau for 12–14 h of treatment, after which it started to decrease until 16 h (Fig. 3). Post 16 h of rifampicin treatment all tested strains started developing adaptive resistance (data not shown).

To confirm whether the isolated persister cells will show similar trajectory of killing curve to their respective initial treatments, we performed re-treatment experiments with vancomycin and vancomycin/rifampicin to represent the single and combination antibiotic treatments respectively. For this, the persister cells harvested after 48- or 72 h were washed in 1X PBS and re-inoculated in fresh MHB. The cells were regrown and treated with respective antibiotics exactly to their initial experiment. These re-treatments continued for four consecutive cycles and CFUs counts from each re-treatment was plotted. We found the identical biphasic killing as well as similar significant differences in persister cells between the wild type and mutant strains to their respective initial experiment results (Additional file 6: Figure S3). These results confirm that the *msaABCR* deletion mutant is defective in persister cells, and the difference in CFUs between the USA300 LAC and the msaABCR deletion mutant is not due to difference in resistance patterns.

### Stationary phase *S. aureus* cells show less tolerance to gentamicin and its combination

**Fig. 5:**
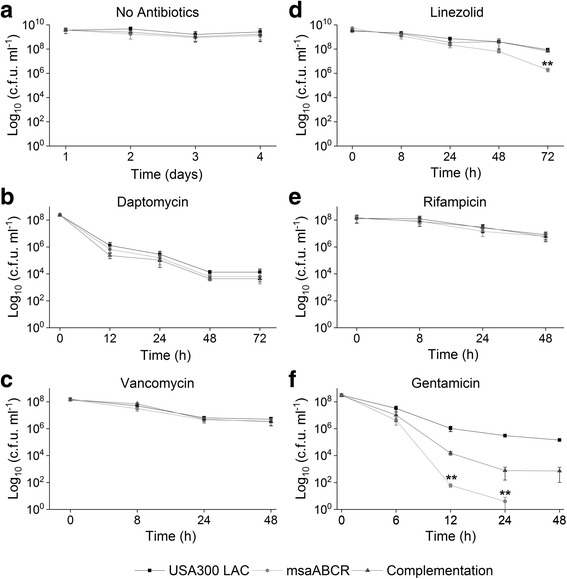
Persister cells from stationary phase growth. Strains USA300 LAC, *msaABCR* mutant, and the complementation were grown to stationary growth phase and then exposed to high concentrations of antibiotics. **a.** no antibiotic control. **B.** daptomycin (80 μg/ml, 80× MIC), **c.** Vancomycin (125 μg/ml, 200× MIC), **d.** Linezolid (100 μg/ml, 20× MIC), **e.** rifampicin (4.8 μg/ml, 80× MIC) or **f.** gentamicin (25 μg/ml, 5× MIC). These results represent the means of three independent experiments. Error bars represent SE. Student’s t-test (OriginPro) was used to compare the results of the wild-type to mutants at respective designated time points (* *p*-value <0.05, ** *p*-value <0.005)

Previous studies have shown that stationary phase *S. aureus* cells are extremely tolerant to conventional drugs behaving as persister cells [10, 65, 66]. We studied the persister cells by all the strains in presence of different antibiotic stress during the stationary growth phase. We observed that both the wild type and *msaABCR* deletion mutant were highly tolerant to vancomycin (200×), and rifampicin (40×) treatment (Fig. 5c and e). Daptomycin (80×) treatment killed most of the stationary phase cells (all three strains tested), and showed smooth killing curve thus forming distinct persister plateau after 48 h of exposure, however, there is no significant difference in the persister cells between USA300 LAC and *msaABCR* deletion mutant (Fig. 5b). Interestingly, the *msaABCR* deletion mutant cells are completely reduced to an undetectable level with gentamicin treatment. Gentamicin (5×) treatment rapidly killed stationary phase *msaABCR* mutant cells within 12 h of treatment, resulting into 1.72X10^4^-fold reduced persister cells relative to wild type. No detectable CFUs were observed in the *msaABCR* deletion mutant after 24 h post-treatment with gentamicin, whereas 5 log of CFUs were still observed in the wild type cells until 48 h of treatment (Fig. 5f). In fact, this is the only antibiotic against which no persister cells were detected in the stationary phase *msaABCR* mutants’ cells. Similarly, the stationary phase *msaABCR* deletion mutants’ cells formed 44.67-fold less persister cells relative to the USA300 LAC when treated with linezolid (20×) for 72 h (Fig. 5d). This contrasts with exponential growth phase cells where linezolid treatment showed no difference between USA300 LAC and *msaABCR* deletion mutants’ persister cells.

**Fig. 6:**
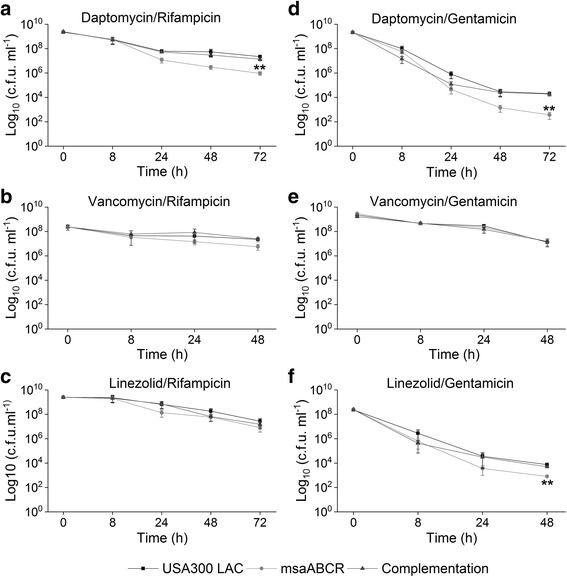
Persister cells from stationary phase growth using combined antibiotics. The cells from stationary growth phase (USA300 LAC, *msaABCR* mutant and the complementation) were treated with combinations of antibiotics. **a.** daptomycin/rifampicin (320× MIC), **b.** vancomycin/rifampicin (40× MIC), **c.** linezolid/rifampicin (160× MIC), **d.** daptomycin/gentamicin (30× MIC), **e.** vancomycin/gentamicin (20× MIC), and **f.** linezolid/gentamicin (20× MIC). These results represent the means of three independent experiments. Error bars represent SE. Student’s t-test (OriginPro) was used to compare the results of the wild-type to mutants at respective designated time points (* *p*-value <0.05, ** *p*-value <0.005)

When we challenged the stationary cells with antibiotic combinations, we observed a pronounced biphasic killing curve dependent on the combination treatments (Fig. 6). The *msaABCR* deletion mutant showed 23-fold reduced persister cells relative to its wild type and complementation strains when treated with daptomycin/rifampicin, but the wild type and complementation strains were not much affected, suggesting the presence of more tolerant cells in these strains (Fig. 6a). Daptomycin/gentamicin combination was found most effective in killing the stationary phase cells and the *msaABCR* deletion mutant showed 54-fold reduced persister cells relative to its wild type and the complementation strains (Fig. 6d). Likewise, linezolid/gentamicin combination was also found most effective in killing the stationary phase cells and reduced the rate of persister cells formation in the *msaABCR* mutant by 9.5-fold (Fig. 6f). However, vancomycin/gentamicin combinations did not show any effect on the stationary phase cells of all strains tested (Fig. 6e). Other antibiotic combinations (vancomycin/rifampicin and linezolid rifampicin) also did not have any difference in the level of persistence between the *msaABCR* deletion mutant and the USA300 LAC strains (Fig. 6b, and c; Table 4). We also tested the persister phenotype after *msaABCR* deletion in Vancomycin Intermediate *S. aureus* (VISA) strain Mu50 in planktonic growth phase (exponential and stationary) after treatment with Daptomycin (10X MIC, 20 μg/ml). Our results showed that deletion of *msaABCR* operon in Mu50 background significantly reduced the persister cells in both exponential and stationary growth phases in presence of daptomycin (Additional file 7: Figure S4). Thus, *msaABCR* operon is involved in persister cells formation in both MRSA and VISA *S. aureus* strain background.

### The *msaABCR* mutant shows reduced persister cells in biofilm

**Fig. 7:**
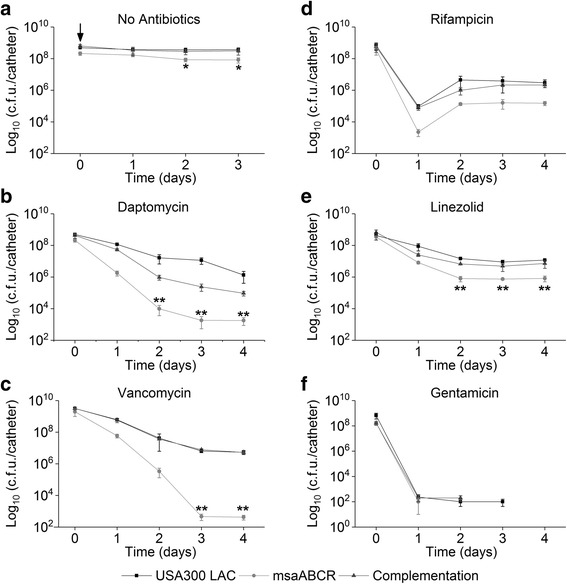
Persister cells on biofilms. Strains USA300 LAC, *msaABCR* deletion mutant, and the complementation were allowed to form biofilm on intravenous catheters for 24 h. Biofilm were then exposed to high concentration of antibiotics. **a.** no antibiotic control, **b.** daptomycin (20 μg/ml, 20× MIC), **c.** vancomycin (12.5 μg/ml, 20× MIC), **d.** rifampicin (4.8 μg/ml, 80× MIC), **e.** linezolid (100 μg/ml, 20× MIC), and **f.** gentamicin (100 μg/ml, 20× MIC). The arrow in the no antibiotic control (**a.**) represents the starting point for the antibiotic treatment in the tests. These results represent the means of three independent experiments performed in triplicate. Error bars represent SE. Student’s t-test and one-way ANOVA (OriginPro) followed by Tukey’s test was used to compare the results of the wild-type to mutants at respective designated time points (* *p*-value <0.05, ** *p*-value <0.005)

**Fig. 8:**
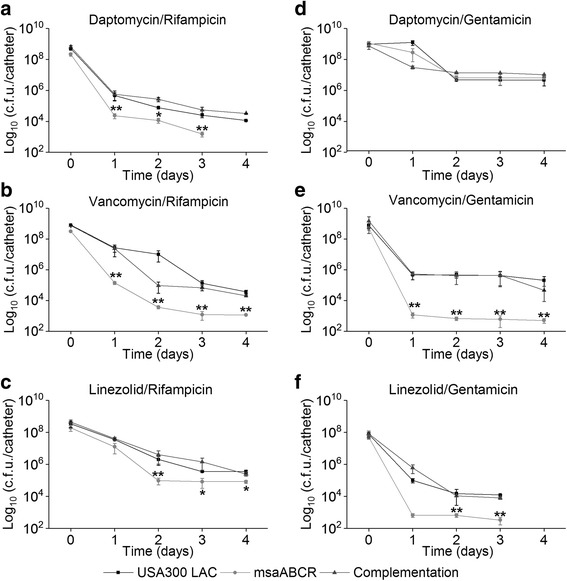
Persister cells from biofilms treated with combinations of antibiotics. Strains USA300 LAC, *msaABCR* deletion mutant, and the complementation were allowed to form biofilm on intravenous catheters for 24 h and then treated with combined antibiotics. **a.** daptomycin/rifampicin (80× MIC), **b.** vancomycin/rifampicin (40× MIC), **c.** linezolid/rifampicin (320× MIC), **d.** daptomycin/gentamicin (40× MIC), **e.** vancomycin/gentamicin (20× MIC), **f.** linezolid/gentamicin (20× MIC). These results represent the means of three independent experiments performed in triplicate. Error bars represent SE. Student’s t-test and one-way ANOVA (OriginPro) followed by Tukey’s test was used to compare the results of the wild-type to mutants at respective designated time points (* *p*-value <0.05, ** *p*-value <0.005)

Biofilms have been shown to be an important source of persister cells [4, 10, 13, 21]. In previous studies, we have shown that *msaABCR* deletion mutant forms weak and loose biofilms, and are defective in late stages of biofilm formation (biofilm maturation). However, they are not defective in early stages of biofilm formation (attachment and accumulation). This led us to speculate that the loose and weak biofilm produced by *msaABCR* deletion mutant may not capable of maintaining persister cells within the biofilm. We measured the rate of persister cell formation in all three strains in intravenous catheter based biofilm conditions. Before studying persister cells in biofilm conditions, we first assessed the biofilm formation by the USA300 LAC, *msaABCR* deletion mutant and the complementation strains on the intravenous catheters for four days without antibiotic treatment. We observed that the *msaABCR* deletion mutant formed 2.3-fold reduced biofilm after day 1 and day 2, and 4.40-fold reduced biofilm after day 3 and day 4, relative to USA300 LAC (Fig. 7a). The number of CFUs on catheter for the USA300 LAC and *msaABCR* deletion mutant were 4.89X10^8^ and 2.13X10^8^ respectively after day 1, and thus used this time point for antibiotic treatment to study the persister cells. (Fig. 7a). We initiated the biofilm formation on the intravenous catheters for 24 h, and then a continuous antibiotic treatment was applied for four days (Figs. 7 and 8). Bacterial CFUs were determined on a subset of catheters each day after antibiotic treatment. Bacterial CFUs were also determined on subset of catheters prior to the initiation of antibiotic treatment (day 0).

We observed pronounced biphasic killing curves in biofilms of all strains when challenged with all antibiotics (individual and combinations) except with rifampicin (40×) treatment, where we observed adaptive resistance after one day of exposure (Fig. 7d). With daptomycin (20×) treatment, the wildtype strain generated 1.34X10^6^ persister cell by the end of four days of continuous treatment, while the *msaABCR* deletion mutant generated significantly fewer persister cell, 1.80X10^3^, thus representing a 747-fold reduction relative to the wildtype strains (Fig. 7b). Vancomycin (20×) treatment produced the highest killing rate in the *msaABCR* deletion mutant biofilm and showed 1.27X10^4^-fold reduction in persister cells relative to USA300 LAC (Fig. 7c). Linezolid (20×) treatment had the least killing effect and generated relatively high rates of persister cell; 8.25X10^5^ in the *msaABCR* deletion mutant and 1.17X10^7^ in USA300 LAC (Fig. 7e). Treatment with gentamicin (20×) generated very less persister cell in both wild type USA300 LAC and *msaABCR* deletion mutant strains after one day of treatment, but interestingly, no detectable persister cells were observed in any of the strains after day 2 of treatment (Fig. 7f).

The antibiotic combinations were found most effective against the *msaABCR* deletion mutants’ biofilm and required significantly less antibiotic concentrations relative to individual antibiotic (Additional file 3: Table S3). We found that after four days of continuous treatment, daptomycin/rifampicin (160×) effectively reduced the number of persister cells generated by the *msaABCR* deletion mutant to an undetectable level relative to USA300 LAC (Fig. 8a). Vancomycin/gentamicin (20×) and vancomycin/rifampicin (40×) also significantly reduced the number of persister cells generated by the *msaABCR* deletion mutant by 407-fold and 32-fold, respectively relative to the USA300 LAC strain after four days of continuous antibiotic treatment (Fig. 8e and b). Linezolid/gentamicin (40×) combination reduced the persister cells from the *msaABCR* deletion mutants’ biofilm by 37.50-fold relative to USA300 strains after day 3 treatment, however, after day 4 treatment, no detectable CFUs were observed in all strains (Fig. 8f). Other antibiotic combinations like linezolid/rifampicin (40×) and daptomycin/gentamicin (40×) treatment resulted into similar fractions of persister cells in all of the strains tested (Fig. 8c and d). Therefore, combined antibiotics treatment increases the killing rate of cells growing in biofilm conditions generating comparatively less persister cells, with *msaABCR* deletion mutant being more susceptible relative to the individual antibiotic treatments. All the results were analyzed with Students t-test as well as with one-way ANOVA followed by Tukey’s HSD test to confirm the results from t-test to effectively evaluate the differences between the wild type and mutants’ biofilm under controlled and antibiotics treatment conditions.

### The *msaABCR* deletion mutant is defective in persister cells under continuous antibiotic treatment condition

We observed significant defect in persister cells formation by the *msaABCR* deletion mutants’ biofilm under static in-vitro catheter based biofilm model. We further confirmed these results by studying the effect of antibiotics treatment in biofilm under continuous flow condition by using BioFlux 200 system (Fluxion Biosciences Inc.) and a 24-well microfluidic plate device*.* Biofilms were formed in BioFlux200 24-well plates and monitored by bright field microscopy every 10 min for 3 days using an inverted microscope (Leica DM IL LED Fluo Inverted Microscope; Leica Microsystems). In the absence of antibiotics, the wildtype and the complementation strains formed similar biofilm. Cells attached to the surface after inoculation (0 h), and biofilm formation increased gradually for 15 h. A rapid accumulation of biofilm was observed at 13 h post-inoculation. After this step, the mature biofilm underwent periodic cycles of sloughing off and maturation every 4–5 h. The *msaABCR* mutant showed similar biofilm growth pattern to wild type USA300 LAC strain till 15 h. After 15 h, the *msaABCR* mutants’ biofilm was significantly dropped to 12.5% compared to that of the wildtype, 76.42% (Additional file 8: Figure S5). This is consistent with our previous studies, which showed that the *msaABCR* deletion mutant fails to develop a mature biofilm [40, 41]. We selected the 12-h time point at which to introduce antibiotics and to examine the effect of continuous antibiotic treatment on biofilm growth and the emergence of any persister cells. We tested the same antibiotics and concentrations as described above for the in vitro catheter-based biofilm assay (Additional file 3: Table S3). Biofilms formed by the *msaABCR* deletion mutant that were treated with a continuous flow of antibiotics (20× of daptomycin, vancomycin, or linezolid) were readily cleared immediately after their introduction relative to USA300 LAC biofilms. However, the *msaABCR* deletion mutant still developed some small colonies after 38 h of post continuous treatment with both daptomycin and linezolid. These colonies persisted until the end of the experiment. Continuous treatment with rifampicin (20×) or gentamicin (20×) showed no impact on any of the strains tested (data not shown). Representative figures for daptomycin and vancomycin treatment are shown in Additional file 9: Figure S6 and Additional file 10: Figure S7 respectively.

We also tested the combination of antibiotics in the Bioflux biofilm model. Continuous daptomycin/rifampicin (160×), vancomycin/rifampicin (40×), and vancomycin/gentamicin (20×) treatments reduced the *msaABCR* deletion mutant biofilm after 12 h to undetectable levels compared with the USA300 LAC biofilm. Continuous linezolid/rifampicin (40×) and linezolid/gentamicin (40×) treatments produced a similar impact in all the strains tested; however, daptomycin/gentamicin (40×) had the least effect on the *msaABCR* deletion mutant relative to USA300 LAC. Representative figures for daptomycin/rifampicin and vancomycin/rifampicin are shown in Additional file 11: Figure S8 and Additional file 12: Figure S9 respectively. The results from treating the biofilms with antibiotics in the Bioflux system were consistent with the in vitro intravenous catheter biofilm results. Therefore, our findings show that the *msaABCR* deletion mutants’ biofilm is unable to persist under treatment by some antibiotic combinations and is easily cleared compared with the wildtype USA300 LAC strain under the same treatment conditions.

### Genes related to formation of persister cells are differentially regulated by *msaABCR*

**Fig. 9:**
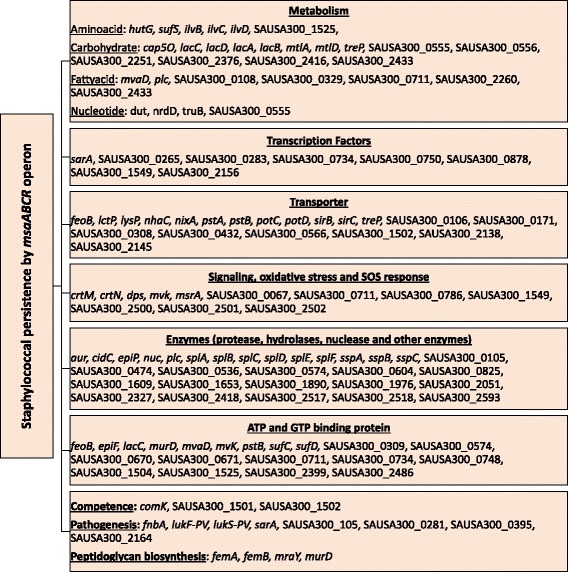
Comparative gene ontology analysis of *msaABCR* transcriptomics by for RNA-sequencing using Illumina- HiSeq 2000. The data was aligned by using Tophat-2.0.8b [50, 51]. Cufflinks-2.1.1 [52, 53] was used to measure the transcript level. Cuffdiff was used to determine expressed transcripts and measure the differential expression of genes in the *msaABCR* deletion mutant compared to wild type USA300 LAC. All the genes that are differentially expressed greater than 3-fold were considered significant and were analyzed by web-based GO analysis tool (Comparative GO) [54]

Previous studies have shown that several metabolic genes, transport, signaling, and oxidative-stress-related genes play role in the formation of persister cells in *S. aureus* [19, 21, 30, 38, 67, 68]. We conducted a genome-wide RNA-seq analysis of the *msaABCR* mutant relative to wild type during the mid-exponential growth phase. We found that 238 genes were differentially expressed in the mutant at 3-fold or higher relative to wild type. Comparative Gene Ontology analysis of *msaABCR* RNA-seq transcriptomics data showed that *msaABCR* operon plays role in the regulation of genes that are involved in metabolisms (amino acids, carbohydrate, fatty acids, nucleotides), transporters, Signaling and oxidative stress, and Enzymes (Proteases, hydrolases and nucleases) (Fig. 9; Additional file 13: Table S4). We found a total of 31 genes that plays role in metabolism were differentially expressed (>3 fold). Of 31 metabolic genes found in our analysis, 14 genes are involved in carbohydrate metabolism, 6 genes are involved in amino acid metabolism, 7 genes are involved in fatty acid metabolism, and 4 genes are involved in nucleotide metabolism (Fig. 9; Additional file 13: Table S4). Deletion of *msaABCR* also affected the expression of 8 transcription factors. Of those, *sarA*, SAUSA300_0265, SAUSA300_0750, SAUSA300_0878, and SAUSA300_2156 play important roles in virulence, cell growth, metabolism, quorum sensing, and biofilm development [48, 69–74]. A total of 20 genes that plays role in transport: phosphate, sodium ion, ferrous ion, nickel/cobalt ion, cation, polyamines, drug, carbohydrate, and amino-acid were affected. Several genes that play roles in cell-signaling, maintaining homeostasis, oxidative stress and SOS response were also affected (Fig. 9; Additional file 13: Table S4). Regulation of enzymes plays important role maintaining the normal physiology of cells. Several enzymes like hydrolases, metalloproteases, serine and cysteine proteases, endoribonucleases, nucleases, oxidases and reductases were also affected by the deletion of *msaABCR* operon. Several ATP and GTP binding proteins and/or enzymes (20 genes) were also affected. A total of four genes that plays an important role in cell-wall biosynthesis (*femA, femB, mraY, and murD*) were affected (Additional file 13: Table S4).

We also studied the transcriptomics of *msaABCR* deletion mutant under biofilm conditions. We found 26 genes that were differentially expressed in the mutant. Three genes that plays role in amino-acid metabolism (*purQ, ald,* and *ilvA*) and the *pur* operon genes (*purC, purD, purE, purF, purH, purK, purL, purM, purN, purQ,* and *purS*) that plays important role in ‘de novo’ IMP biosynthetic process were affected. Others genes that plays role in cell death (*lrgA* and *lrgB*), transmembrane transport, PTS system (mannitol specific IIBC component), metalloaminopeptidase, formate dehydrogenase, and Oxygen-dependent choline dehydrogenase were also affected (Additional file 14: Table S5).

These findings suggest that the *msaABCR* deletion mutant is defective in regulating genes that are important for metabolism, proteases and hydrolases enzymes, transporter systems and other global regulators that may play roles in maintaining dormancy during the persister stage. In the future, we plan to further examine the direct or indirect regulation of these genes by *msaABCR* operon and define a mechanistic role during persister cells formation.

## Discussion

Antibiotic treatment failures are becoming increasingly problematic, and potential solutions can range from finding new antibiotics to improving the effectiveness of existing ones. Several chronic infections caused by *S. aureus* are difficult to treat because of the emergence of persister cells, which are genetically sensitive to antibiotics but are not killed by them [10, 16, 75]. However, the mechanism for this antibiotic tolerance and persister cells formation is still not well understood, especially in *S. aureus*. Previously, our laboratory characterized the *msaABCR* operon and showed that it plays a key role in virulence, autolysis and biofilm development [40, 41]. We have also shown that *msaABCR* operon increased the susceptibility of VISA strains to vancomycin and is involved in cell wall synthesis [42]. More recently we showed that MsaB, the protein produced from *msaABCR* operon is a transcription factor, that binds its target DNA in response to nutrient availability [39]. These findings led to this current study to examine the role of the *msaABCR* operon in the *S. aureus* persister cells formation in presence of antibiotics stress since persister cells formation is related to nutrient response to some extent. Here, we show that deletion of the *msaABCR* operon in USA300 LAC not only increased the efficiency of drugs-combination but also formed significantly less number of persister cells in planktonic as well as biofilm growth conditions. Importantly, the concentration of antibiotics used in the combination treatments, which significantly impacted both persister cell formation and biofilm viability in the *msaABCR* deletion mutant, are within the concentrations defined by the CLSI as the breakpoint MICs for MRSA strains of *S. aureus.*

This study demonstrates the role of the *msaABCR* operon in persister cells development and survival in *S. aureus* under antibiotic stress. This adds a new regulator into the limited body of knowledge about the mechanism of persister cells formation in *S. aureus*. We showed that the deletion of the *msaABCR* operon significantly reduces the rate of persister cell formation in all growth phases: exponential, stationary, and biofilm. The trajectories of killing curves and formation of characteristic persister plateau observed was dependent on growth phases and the particular antibiotics used (individual or combinations). This may be because previous studies have shown the presence of physiologically heterogeneous groups of populations among the cells of *S. aureus* [63, 64]. For instance, during exponential growth phase, all individual and combined antibiotic tested showed good trajectories of killing curves and the *msaABCR* deletion mutant formed significantly less number of persister cells relative to wild type. However, linezolid, linezolid/rifampicin and linezolid/gentamicin did not show any significant difference between wild type and mutant strains, and showed high number of robust persister cells in all strains tested. The re-treatment experiment in which the persister fraction obtained was re-inoculated in fresh media and again treated with the antibiotics showed similar trajectory of killing kinetics confirms that remaining cells after the initial treatments are persister cells and not resistant cells.

Consistent with previous studies related to persister cells in *S. aureus*, we also observed significant number of cells that are extremely tolerant to antibiotic treatment during the stationary growth phases. We found most of the antibiotic treatment (individual or combinations) used in this study generated significant number of persister cells during stationary growth phase. Daptomycin treatment killed most of the stationary phase cells; however, the numbers of persister cells are similar in all strains. Importantly, gentamicin treatment reduced the persister cells in the *msaABCR* deletion mutant to an undetectable limit within 12 h, whereas robust persister cells are still present until 48 h in the USA300 LAC. Furthermore, daptomycin/gentamicin and linezolid/gentamicin treatments were also found very effective in killing stationary phase *S. aureus* cells, with the *msaABCR* deletion mutant being killed more and thus resulting into significant drop in persister cells in the mutant relative to USA300 LAC and complementation strains. In addition, significant reduction in persister cells in the *msaABCR* deletion mutant in VISA strain (Mu50) background when treated with daptomycin under both exponential and stationary growth phases suggest that this effect is strain independent. However, more strains need to be analyzed to ensure that the role of the *msaABCR* operon in formation of persister cells is conserved in *S. aureus*.

In biofilm growth conditions, we observed the pronounced effect of *msaABCR* operon deletion. The mutants’ biofilm was severely defective in persister cells by several orders of magnitude compared to the USA300 LAC with all antibiotics treatment. However, with rifampicin treatment, we observed increased resistance patterns after day 1 treatment. A similar phenomenon was observed by Raad et al. [76], who found that treatment with rifampicin alone is not sufficient for reducing the microbial burden in catheter-associated biofilms and that such treatment is associated with the emergence of rifampicin resistance in MRSA strains. The same study also showed that rifampicin in combination with other antibiotics is successful in eliminating biofilms [76]. We reasoned that the defective biofilm in the *msaABCR* deletion mutant, characterized by loosely connected matrix may not be favorable to formation of persister cells. However, further studies are needed to understand these observations or the mechanism used by *msaABCR* in antibiotic tolerance.

Mechanism of formation of persister cells has been studied well in *E. coli*, *pseudomonas*, and *Mycobacterium tuberculosis* but relatively little is known about mechanisms in *S. aureus*. Yee et al. [38] recently studied the role of metabolism (purine, amino acid, carbohydrate, Lipid, vitamins), proteases and hydrolases, and transporters in the persistence in presence of rifampicin. Conlon et al. [19] showed that depletion of intracellular ATP is important for the *S. aureus* persister cell formation. In USA500 strains, genes essential in TCA cycle, such as *sdhA*, *sdhB* and *sdhC*, were found to reduce the persister cells [77]. Small colony variants (SCVs) in *S. aureus* were found more tolerant to the antibiotics due to defective electron transport chain specifically heme and menaquinone synthesis deficiency [78]. In addition, pathways activated in SCVs were also upregulated in persister cells [79], which suggest that changes in metabolic pathways lead to slow-growth and consequent persistence in *S. aureus*. Indeed, persistence appears to be the extensively dependent on bacterial metabolism [31, 67, 80]. All these results indicate the importance of metabolic processes, energy production, and transporter genes etc. in the persister development and survival in *S. aureus*.

Our transcriptomics data suggest that the *msaABCR* operon may control the expression of genes that may be involved in the formation of persister cells. Under planktonic growth condition, we showed that this operon affects the expression of several genes including transcription factors, metabolic genes, transporters genes, ATP and GTP binding protein encoding genes and cell wall biosynthetic genes. Several genes that have been previously linked with persister cells formation in other bacterial systems like superoxide dismutase [Mn/Fe]_2_, iron-sulfur cluster assembly scaffold protein NifU, pyruvate oxidase, ketol-acid reducto-isomerase, phosphate ABC transporter/permease protein PstA, serine protease, cysteine proteases and metalloproteases were also affected by the deletion of *msaABCR* operon [10, 30, 31, 36, 67, 81–83]. Under the biofilm growth conditions, the *pur* operon was differentially expressed in the *msaABCR* mutant. Previously, Yee et al. [38] showed that the *pur* operon plays role in persister cells formation in presence of rifampicin treatment in *S. aureus*. Additionally, defective purine biosynthesis has been linked to the defective biofilm formation in other bacterial systems like *Streptococcus sanguinis* [84] and in Burkholderia [85]. The defective and immature biofilm formation in *msaABCR* deletion mutant is mediated through the increased processing of major autolysin, Atl, by excessive protease production which led to uncontrolled cell death. However, further studies are needed to determine whether the excessive protease production also resulted in the defect of formation of persister cells in the *msaABCR* mutant. Likewise, whether the *msaABCR* operon plays role in formation of persister cells regulating the purine genes also remains to be elucidated. Other genes that plays role in amino-acid metabolism, programmed cell death, transmembrane transport and metalloaminopeptidase were also affected in *msaABCR* mutants’ biofilm. However, these findings are just the initial steps in defining the mechanism of persister cell formation in *S. aureus* by *msaABCR* operon and further studies are needed to define the mechanistic role of the *msaABCR* operon in the *S. aureus* antibiotics response and/or persistence.

Overall, these results suggest that targeting the *msaABCR* operon leads to reduced formation of persister cells in MRSA strains in both planktonic and biofilm growth conditions. More importantly, the concentration of antibiotics in the combination treatments, that significantly impacted both persister cell formation and biofilm viability in the *msaABCR* deletion mutant, are within the concentrations defined by the CLSI as the breakpoint MICs for MRSA strains of *S. aureus.* Thus, inactivation of the *msaABCR* operon might enhance the effectiveness of antibiotics used in the treatment of MRSA infections, especially in context of persister cells.

## Conclusions

In this study, we have shown that *msaABCR* operon plays role in the persister cells formation in *S. aureus.* We have also shown that the persister cell formation in *S. aureus* is dependent on the growth phases (exponential, stationary, and biofilm) and type of antibiotic treatment applied (Individual Vs Combined). This is evident by the different trajectories of killing curves generated by different antibiotics treatment under different growth conditions. Stationary and biofilm growth conditions of *S. aureus* are known to the most favorable physiological niche for the persister cells and presumed to be very tolerant to most traditional drugs. Importantly, we have shown that deletion of *msaABCR* operon significantly affected the persister cells formation under stationary growth phases with antibiotics treatment like gentamicin, daptomycin/gentamicin and linezolid/gentamicin. In addition, the *msaABCR* deletion mutant is severely defective in persister cells formation under biofilm growth conditions when treated with almost all individual and combined antibiotics, except rifampicin and daptomycin/gentamicin treatment. We also showed that deletion of *msaABCR* operon regulates the expression of several genes that are important in metabolism, virulence, transports, cell wall metabolism and several transcription factors. However, we have not yet defined the exact mechanism by which *msaABCR* exerts its role in persister cells formation, but we seek to define the mechanism in future. Thus, this study provides additional insight into a limited body of knowledge about the mechanism of persistence in *S. aureus*.

## Additional files

Additional file 1: Table S1. Concentrations of antibiotics used to study persister cells from exponential growth phase (DOCX 15 kb)

Additional file 2: Table S2. Concentrations of antibiotics used to study persister cells from stationary growth phase (DOCX 15 kb)

Additional file 3: Table S3. Concentrations of antibiotics used to study persister cells from biofilm (DOCX 15 kb)

Additional file 4: Figure S1. Growth kinetics at exponential and stationary growth phase. The bacterial cells (USA300 LAC, *msaABCR* deletion mutant and complementation) were grown in MHB media. The cells were normalized to OD600 of 0.05 before measuring the growth kinetics. The graph showed no observable growth defect among the strains tested in both a. exponential and b. stationary phase. These results represent the means of three independent experiments run in triplicates. Error bars represent SE. Student’s t-test (OriginPro) was used to compare the results of the wild-types to their respective mutants (* *p*-value <0.05, ** p-value <0.005) (TIFF 596 kb)

Additional file 5: Figure S2. Control biofilm formation on the MBEC pegs without antibiotics after 24 h of incubation. The MBEC pegs were coated with 20% human plasma and biofilms were allowed to in the pegs for 24 h. The *msaABCR* mutant strains formed 2.23-fold reduced biofilm relative to wild type strains. These results represent the means of three independent experiments run in triplicates. Error bars represent SE. Student’s t-test and one-way ANOVA (OriginPro) followed by Tukey’s test was used to compare the results of the wild-type to mutants (* *p*-value <0.05, ** p-value <0.005) (TIFF 605 kb)

Additional file 6: Figure S3. Re-treatment assay for persister cells. The cells (USA300 LAC, *msaABCR* mutant, and complementation) growing exponentially were treated with a. vancomycin (40× MIC) or b. vancomycin/rifampicin (20× MIC). After treatment, the surviving persister cells were reinoculated in fresh medium, cultured and retreated with antibiotics. The re-treatment experiment was performed four consecutive times. RT denotes re-treatment. Student’s t-test (OriginPro) was used to compare the results of the wild-type to mutants at their respective end points (* *p*-value <0.05, ** *p*-value <0.005) (TIFF 430 kb)

Additional file 7: Figure S4. Persister assay in Mu50 strain background. Strains Mu50, *msaABCR* mutant and the complementation strains were grown to a. exponential and b. stationary growth phase. Strains were then exposed to daptomycin (20 μg/ml, 10× MIC). Time 0 h represents antibiotic exposure point. These results represent the means of three independent experiments. Error bars represent SE. Student’s t-test (OriginPro) was used to compare the results of the wild-type to mutants at respective designated time points (* *p*-value <0.05, ** *p*-value <0.005) (TIFF 167 kb)

Additional file 8: Figure S5. Biofilm formation in continuous flow in absence of antibiotics (control). Strains USA300 LAC, *msaABCR* deletion mutant and the complementation were inoculated into the BioFlux microfluidic system and allowed to form a biofilm. The media flow rate was set at 64 μl/h and biofilm was monitored for up to 50 h. Bright-field images were collected at 10 m intervals using an inverted Leica DM IL LED Fluo Inverted Microscope under 5X magnification. a. Area coverage of biofilm. b. Representative images of biofilm development at different time points (TIFF 9454 kb)

Additional file 9: Figure S6. Continuous monitoring of biofilm formation in the presence of daptomycin. Stains USA300 LAC, *msaABCR* deletion mutant and the complementation were inoculated into the BioFlux microfluidic system and allowed to form a biofilm for 12 h. Daptomycin (20 μg/ml, 20× MIC) treatment was started and the bright field images were collected at 10 m intervals using an inverted Leica DM IL LED Fluo Inverted Microscope under 5X magnification. The 0-h time point indicated the point of antibiotic treatment. The flow rate of media containing daptomycin was set at 64 μl/h and the biofilm was monitored up to 50 h. a. Area coverage of biofilm. b. Representative images of biofilm development at different time points (TIFF 9480 kb)

Additional file 10: Figure S7. Continuous monitoring of biofilm formation in the presence of vancomycin. Stains USA300 LAC, *msaABCR* deletion mutant and the complementation were inoculated into the BioFlux microfluidic system and allowed to form a biofilm for 12 h. Vancomycin (12.50 μg/ml, 20× MIC) treatment was started and 0-h time indicated the point of antibiotic treatment. The flow rate of media with vancomycin was set at 64 μl/h and biofilm was monitored up to 50 h. Bright-field images were collected every 10 m intervals using an inverted Leica DM IL LED Fluo Inverted Microscope under 5X magnification. a. Area coverage of biofilm. b. Representative images of biofilm development at different time points (TIFF 9042 kb)

Additional file 11: Figure S8. Continuous monitoring of biofilm formation in the presence of daptomycin/rifampicin. Stains USA300 LAC, *msaABCR* deletion mutant and the complementation were inoculated into the BioFlux microfluidic system and allowed to form a biofilm for 12 h. Daptomycin/Rifampicin (160× MIC) combinations treatment was started and bright-field images were collected at every 10 m intervals using an inverted Leica DM IL LED Fluo Inverted Microscope under 5X magnification. The media with antibiotics flow rate was set at 64 μl/h and biofilm was monitored up to 50 h. a. Area coverage of biofilm. b. Representative images of biofilm development at different time points (TIFF 10521 kb)

Additional file 12: Figure S9. Continuous monitoring of biofilm formation in the presence of vancomycin/rifampicin. Stains USA300 LAC, *msaABCR* deletion mutant and the complementation were inoculated into the BioFlux microfluidic system and allowed to form a biofilm for 12 h. Vancomycin/Rifampicin (40× MIC) combinations treatment was started and bright-field images were collected at every 10 m intervals using an inverted Leica DM IL LED Fluo Inverted Microscope under 5X magnification. The 0 h time point indicated the start of antibiotic treatment. The flow rate of antibiotic treatment was set at 64 μl/h and biofilm was monitored for up to 50 h. a. Area coverage of biofilm. b. Representative images of biofilm development at different time points (TIFF 10570 kb)

Additional file 13: Table S4. Comparative gene ontology analysis of *msaABCR* transcriptomics under planktonic growth phase (DOCX 23 kb)

Additional file 14: Table S5. Comparative gene ontology analysis of *msaABCR* transcriptomics under biofilm growth conditions (DOCX 16 kb)

## Abbreviations

°C: Degree Celsius
AD: additive
AN: antagonistic
Atl: Major autolysin
Biofilm media: TSB supplemented with 3% sodium chloride and 0.5% glucose
CaCl_2_: Calcium chloride
CA-MRSA: Community Acquired Methicillin Resistant *Staphylococcus aureus*
CFUs: Colony forming units
CLSI: Clinical and Laboratory Standards Institute
DAP: Daptomycin
FEP: Fluorinated ethylene propylene
FIC index: Fractional inhibitory concentration index value
GEN: Gentamicin
GO: Gene ontology
h: Hour
IN: Indifference
INT: Interpretation
LIN: Linezolid
Log: Logarithmic
m: Minutes
MBEC: Minimum biofilm eradication concentration
MHB: Mueller-Hinton broth
MIC: Minimum inhibitory concentration
MRSA: Methicillin Resistant *Staphylococcus aureus*
OD: Optical density
PBS: Phosphate-buffered saline
RIF: Rifampicin
RNA-seq: RNA sequencing
Rpm: Rotation per minute
SE: Standard error
SN: synergy
TCA: Tricarboxylic acid cycle
TSA: Tryptic soy agar
TSB: Tryptic soy broth
UD: undetectable
VAN: Vancomycin
μl: microliter
