# Supplementary material for: msaABCR operon is involved in persister cell formation in Staphylococcus aureus
Source: BMC Microbiol. 2017 Nov 22;17:218. doi: 10.1186/s12866-017-1129-9 (PMC5700755; doi:10.1186/s12866-017-1129-9)
Supplement: Supplementary file 1 — Concentrations of antibiotics used to study persister cells from exponential growth phase (DOCX 15 kb) [file 12866_2017_1129_MOESM1_ESM.docx]

**Table S1** Concentrations of antibiotics used to study persister cells from exponential growth phase

| **Individual antibiotics** | | | |
| --- | --- | --- | --- |
| **Antibiotics** | **Concentrations (μg/ml)** | | **Times MIC (X)** |
| DAP | 10 | | 10 |
| VAN | 25 | | 40 |
| RIF | 2.4 | | 40 |
| LIN | 50 | | 10 |
| GEN | 20 | | 4 |
| **Combined antibiotics** | | | |
| **Combination** | **Individual Concentration (μg/ml)** | | **Combined MIC (X)** |
| DAP/RIF | DAP (1.248) | RIF (0.312) | 40 |
| VAN/RIF | VAN (6.25) | RIF (0.074) | 20 |
| LIN/RIF | LIN (3.12) | RIF (0.074) | 20 |
| DAP/GEN | DAP (6.24) | GEN (25) | 20 |
| VAN/GEN | VAN (6.24) | GEN (25) | 20 |
| LIN/GEN | LIN (6.24) | GEN (25) | 20 |

DAP: daptomycin, VAN: vancomycin, RIF: rifampicin, LIN: linezolid, GEN: gentamicin
