# Supplementary material for: msaABCR operon is involved in persister cell formation in Staphylococcus aureus
Source: BMC Microbiol. 2017 Nov 22;17:218. doi: 10.1186/s12866-017-1129-9 (PMC5700755; doi:10.1186/s12866-017-1129-9)
Supplement: Supplementary file 2 — Concentrations of antibiotics used to study persister cells from stationary growth phase (DOCX 15 kb) [file 12866_2017_1129_MOESM2_ESM.docx]

**Table S2** Concentrations of antibiotics used to study persister cells from stationary growth phase

| **Individual antibiotics** | | | |
| --- | --- | --- | --- |
| **Antibiotics** | **Concentration (μg/ml)** | | **Times MIC (X)** |
| DAP | 80 | | 80 |
| VAN | 125 | | 200 |
| RIF | 4.8 | | 80 |
| LIN | 100 | | 20 |
| GEN | 25 | | 5 |
| **Combined antibiotics** | | | |
| **Combination** | **Individual Concentration (μg/ml)** | | **Combined MIC (X)** |
| DAP/RIF | DAP (10) | RIF (2.5) | 320 |
| VAN/RIF | VAN (6.25) | RIF (0.148) | 40 |
| LIN/RIF | LIN (50) | RIF (1.184) | 160 |
| DAP/GEN | DAP (10) | GEN (40) | 32 |
| VAN/GEN | VAN (6.24) | GEN (25) | 20 |
| LIN/GEN | LIN (31.25) | GEN (50) | 20 |

DAP: daptomycin, VAN: vancomycin, RIF: rifampicin, LIN: linezolid, GEN: gentamicin
