# Supplementary material for: msaABCR operon is involved in persister cell formation in Staphylococcus aureus
Source: BMC Microbiol. 2017 Nov 22;17:218. doi: 10.1186/s12866-017-1129-9 (PMC5700755; doi:10.1186/s12866-017-1129-9)
Supplement: Supplementary file 3 — Concentrations of antibiotics used to study persister cells from biofilm (DOCX 15 kb) [file 12866_2017_1129_MOESM3_ESM.docx]

**Table S3** Concentrations of antibiotics used to study persister cells from biofilm

| **Individual antibiotics** | | | |
| --- | --- | --- | --- |
| **Antibiotics** | **Concentration (μg/ml)** | | **Times MIC (X)** |
| DAP | 20 | | 20 |
| VAN | 12.5 | | 20 |
| RIF | 4.8 | | 80 |
| LIN | 100 | | 20 |
| GEN | 100 | | 20 |
| **Combined antibiotics** | | | |
| **Combination** | **Individual Concentration (μg/ml)** | | **Combined MIC (X)** |
| DAP/RIF | DAP (5) | RIF (1.248) | 160 |
| VAN/RIF | VAN (6.25) | RIF (0.148) | 40 |
| LIN/RIF | LIN (6.25) | RIF (0.148) | 40 |
| DAP/GEN | DAP (12.48) | GEN (50) | 40 |
| VAN/GEN | VAN (6.24) | GEN (25) | 20 |
| LIN/GEN | LIN (12.5) | GEN (50) | 40 |

DAP: daptomycin, VAN: vancomycin, RIF: rifampicin, LIN: linezolid, GEN: gentamicin
