# Supplementary material for: msaABCR operon is involved in persister cell formation in Staphylococcus aureus
Source: BMC Microbiol. 2017 Nov 22;17:218. doi: 10.1186/s12866-017-1129-9 (PMC5700755; doi:10.1186/s12866-017-1129-9)
Supplement: Supplementary file 13 — Comparative gene ontology analysis of msaABCR transcriptomics under planktonic growth phase (DOCX 23 kb) [file 12866_2017_1129_MOESM13_ESM.docx]

**Table S4** Comparative gene ontology analysis of *msaABCR* transcriptomics under planktonic growth phase

| **Pathways Involved** | **Mean Fold change** | **Genes Involved** |
| --- | --- | --- |
| **Amino acid Metabolism** | | |
| Histidine catabolic process to glutamate and formamide/formate | -7.37 | hutG |
| Glycyl-tRNA aminoacylation | -4.73 | SAUSA300_1525 |
| Cysteine metabolic process | -3.44 | sufS |
| Isoleucine and valine biosynthetic process | 3.06 | ilvB, ilvC, ilvD |
| **Carbohydrate metabolism** | | |
| Glycerol-3-phosphate catabolic process | -4.34 | SAUSA300_2251 |
| Polysaccharide biosynthetic process | -3.56 | cap5O, SAUSA300_2376 |
| Mannitol metabolic process | -3.49 | mtlD |
| Lactose catabolic process via tagatose-6-phosphate | 4.18 | lacC, lacD |
| Galactose/lactose catabolic process | 6.18 | lacA, lacB |
| Formaldehyde assimilation via ribulose monophosphate cycle | -4.75 | SAUSA300_0555 |
| Phosphoenolpyruvate-dependent sugar phosphotransferase system | -3.68 | treP, mtlA |
| phosphoglucomutase activity | -3 | SAUSA300_2433 |
| carbohydrate derivative metabolic process | -4.76 | SAUSA300_0556 |
| glucose 1-dehydrogenase like protein | -6.127 | SAUSA300_2416 |
| **Fatty acid Biosynthesis** | | |
| phospholipid biosynthetic process | -5.03 | SAUSA300_0711 |
| steroid biosynthetic process | -4.71 | SAUSA300_0329 |
| fatty acid metabolic process | -3.35 | SAUSA300_0108 |
| phosphatidylinositol phosphorylation | -3.06 | SAUSA300_2260 |
| lipid metabolic process | 3.7 | plc |
| isopentenyl diphosphate biosynthetic process, mevalonate pathway | -4.68 | mvaD |
| Glucolipid metabolism | -3 | SAUSA300_2433 |
| **Nucleotides metabolism** | | |
| 'de novo' pyrimidine nucleobase biosynthetic process | -4.75 | SAUSA300_0555 |
| dUTP metabolic process | 3.65 | dut |
| Pseudouridine biosynthesis | -7.727 | truB |
| DNA replication (nucleotide metabolism) | -3.71 | nrdD |
| **Transcription factor** | | |
| transcription, DNA-templated (downregulated) | -3.24 | sarA, SAUSA300_0265, SAUSA300_0750, SAUSA300_0878 |
| transcription, DNA-templated (upregulated) | 3.57 | SAUSA300_0283, SAUSA300_2156 |
| Other DNA binding protein | 10.2 | SAUSA300_0734, SAUSA300_1549 |
| **ATP and GTP binding protein** | | |
| ATPase activity, ABC transporter ATP-binding protein | -6.316 | epiF, SAUSA300_0309, SAUSA300_2399, sufC, sufD |
| ATP binding protein (down regulated) | -5.45 | epiF, murD, mvaD, mvK, pstB, SAUSA300_0309, SAUSA300_0574, SAUSA300_0711, SAUSA300_0748, SAUSA300_1525, SAUSA300_2399, SAUSA300_2486, sufC |
| ATP binding protein (up regulated) | 5.144 | lacC, SAUSA300_0670, SAUSA300_0671, SAUSA300_0734, SAUSA300_1504 |
| GTP binding protein | -3.65 | feoB, SAUSA300_0748 |
| **Transporter** | | |
| phosphate ion transmembrane transport | -4.28 | pstA, pstB |
| protein secretion by the type II secretion system | 12.25 | SAUSA300_1502 |
| polyamine transport | -4.71 | potD |
| Glycine betaine transporter | -11.89 | SAUSA300_2145 |
| Iron compound ABC transporter | -3.86 | sirB, sirC |
| neurotransmitter: sodium symporter | 6.028 | SAUSA300_0432 |
| Cation efflux family protein | -4.2 | SAUSA300_0171 |
| Ferrous iron transport protein B | -4.11 | feoB |
| Nickel/cobalt efflux system | -4.61 | nixA |
| lactate permease | -3 | lctP |
| Amino acid permease | -4.07 | lysP, SAUSA300_0566 |
| Na+/H+ antiporter | -4.64 | nhaC |
| siderophore transmembrane transporter | -5.23 | SAUSA300_2138 |
| PTS system, trehalose-specific IIBC component | -3.87 | treP |
| Spermidine/putrescine ABC transporter | -4.713 | potC, potD |
| Putative drug transporter | -5.14 | SAUSA300_0106 |
| ABC transporter, permease protein | -5.77 | SAUSA300_0308 |
| **Signaling, oxidative stress and SOS response** | | |
| protein kinase C-activating G-protein coupled receptor signaling pathway | -5.03 | SAUSA300_0711 |
| carotenoid biosynthetic process | -9.86 | crtM, crtN, SAUSA300_2500, SAUSA300_2501, SAUSA300_2502, |
| isoprenoid biosynthetic process | -5.13 | mvk |
| response to oxidative stress | -12.87 | msrA, SAUSA300_0786 |
| response to stress | -5 | dps, SAUSA300_0067 |
| DNA repair | 13.7 | SAUSA300_1549 |
| **Enzymes (protease, hydrolases, nuclease and other enzymes)** | | |
| Hydrolase, alpha/beta hydrolase fold family | 5.6967 | SAUSA300_0604 |
| Type III leader peptidase family protein | 5.122 | SAUSA300_1609 |
| Zinc metalloproteinase aureolysin | 28.497 | aur |
| Serine proteases | 17.32 | splA, splB, splC, splD, splE, splF, sspA |
| Lantibiotic epidermin leader peptide processing serine protease | -6.79 | epiP |
| cysteine-type protease | 29.12 | SAUSA300_1890, sspB, sspC |
| Putative succinyl-diaminopimelate desuccinylase | -7.765 | SAUSA300_1976 |
| Peptidase, M20/M25/M40 family | -4.688 | SAUSA300_0105 |
| Hydrolases family protein | -7.064 | SAUSA300_1653, SAUSA300_2517, SAUSA300_2518 |
| Putative transglycosylase SceD | 8.72 | SAUSA300_2051 |
| Putative succinyl-diaminopimelate desuccinylase | -7.76 | SAUSA300_1976 |
| Alkyl hydroperoxide reductase | -6.56 | SAUSA300_2418 |
| pyruvate oxidase | -3.482 | cidC |
| phosphoric diester hydrolase | 3.701 | plc |
| Protein deglycase HchA | -3.48 | SAUSA300_0536 |
| Nuclease | 5.9085 | nuc |
| Putative endoribonuclease L-PSP | -6.462 | SAUSA300_0474 |
| Phosphomevalonate kinase | -4.882 | SAUSA300_0574 |
| Putative pyridoxamine-phosphate oxidase | -5.19 | SAUSA300_2327 |
| Probable nitronate monooxygenase | -5.333 | SAUSA300_0825 |
| Putative riboflavin reductase | -7.11 | SAUSA300_2593 |
| **Others** | | |
| **Establishment of competence** | 10.95 | comK, SAUSA300_1501, SAUSA300_1502 |
| **pathogenesis (down-regulated)** | -6.79 | fnbA, sarA, SAUSA300_0395, SAUSA300_2164 |
| **pathogenesis (up-regulated)** | 3.07 | lukF-PV, lukS-PV, SAUSA300_0281, SAUSA300_1058 |
| **Peptidoglycan biosynthesis** | -3.38 | femA, femB, mraY, murD |
| **Integral component of membrane (Down-regulated)** | -5.717 | feoB, lysP, mraY, nhaC, nixA, potC, SAUSA300_0106, SAUSA300_0171, SAUSA300_0308, SAUSA300_0374, SAUSA300_0566, SAUSA300_1685, SAUSA300_1864, SAUSA300_2145, SAUSA300_2376, SAUSA300_2500, SAUSA300_2502, sirB, sirC, treP |
| **Integral component of membrane (Up-regulated)** | 4.03 | SAUSA300_0180, SAUSA300_0205, SAUSA300_0432, SAUSA300_0670, SAUSA300_0671, SAUSA300_2289 |

Comparative GO analysis of the RNAseq transcriptomics data of *msaABCR* deletion mutant. All the genes that are differentially expressed greater than 3-fold in the *msaABCR* deletion mutant relative to wild type was considered significant and were analyzed by web-based GO analysis tool (Comparative GO) [51].
