# Supplementary material for: msaABCR operon is involved in persister cell formation in Staphylococcus aureus
Source: BMC Microbiol. 2017 Nov 22;17:218. doi: 10.1186/s12866-017-1129-9 (PMC5700755; doi:10.1186/s12866-017-1129-9)
Supplement: Supplementary file 14 — Comparative gene ontology analysis of msaABCR transcriptomics under biofilm growth conditions (DOCX 16 kb) [file 12866_2017_1129_MOESM14_ESM.docx]

**Table S5** Comparative gene ontology analysis of *msaABCR* transcriptomics under biofilm growth condition

| **Pathways Involved** | **Average fold change** | **Gene Involved** |
| --- | --- | --- |
| **Aminoacid Metabolism** | | |
| glutamine metabolic process | -4.45 | purQ, |
| Alanine dehydrogenase | 3 | ald |
| L-threonine dehydratase | 3 | ilvA |
|  |  |  |
| **Nucleotide metabolism** | | |
| 'de novo' IMP biosynthetic process | -4.15 | purC, purD, purE, purF, purH, purK, purL, purM, purN, purQ, purS |
| **Others** | | |
| pathogenesis | 3.15 | SAUSA300_1068, SAUSA300_1327 |
| programmed cell death | 3.2 | SAUSA300_0256, SAUSA300_0257 |
| Transmembrane transport | 3 | uhpT |
| PTS system, mannitol specific IIBC component | -3.15192 | mtlF |
| metalloaminopeptidase activity |  | map, |
| Formate dehydrogenase | -3 | SAUSA300_0179 |
| Oxygen-dependent choline dehydrogenase | -3 | betA |
| Phi77 ORF106-like protein | -3 | SAUSA300_1927 |
| Phi77 ORF002-like protein, phage minor structural protein | -3 | SAUSA300_1928 |
| Uncharacterized protein | 3.62 | SAUSA300_0806, SAUSA300_1209 |

Comparative GO analysis of the RNAseq transcriptomics data of *msaABCR* deletion mutant. All the genes that are differentially expressed greater than 3-fold in the *msaABCR* deletion mutant relative to its wild type was considered significant and were analyzed by web-based GO analysis tool (Comparative GO) [51].
